# Retrospective comparison of percutaneous balloon compression and radiofrequency-thermocoagulation in the management of trigeminal neuralgia

**DOI:** 10.1007/s00701-023-05656-w

**Published:** 2023-06-07

**Authors:** Johannes Herta, Theresa Bettina Loidl, Tobias Schmied, Matthias Tomschik, Farjad Khalaveh, Wei-Te Wang, Christian Dorfer

**Affiliations:** Department of Neurosurgery, Medical University of Vienna, Währinger Gürtel 18-20, 1090 Vienna, Austria

**Keywords:** Percutaneous balloon compression, Radiofrequency-thermocoagulation, Trigeminal neuralgia, Facial pain, Outcome analysis, Ablative procedures

## Abstract

**Purpose:**

To compare percutaneous balloon compression (PBC) and radiofrequency thermocoagulation (RFTC) for the treatment of trigeminal neuralgia.

**Methods:**

This was a retrospective single-center analysis of data from 230 patients with trigeminal neuralgia who underwent 202 PBC (46%) and 234 RFTC (54%) from 2002 to 2019. Comparison of demographic data and trigeminal neuralgia characteristics between procedures as well as assessment of 1) initial pain relief by an improved Barrow Neurological Institute (BNI) pain intensity scale of I–III; 2) recurrence-free survival of patients with a follow-up of at least 6 months by Kaplan-Meier analysis; 3) risk factors for failed initial pain relief and recurrence-free survival by regression analysis; and 4) complications and adverse events.

**Results:**

Initial pain relief was achieved in 353 (84.2%) procedures and showed no significant difference between PBC (83.7%) and RFTC (84.9%). Patients who suffered from multiple sclerosis (odds ratio 5.34) or had a higher preoperative BNI (odds ratio 2.01) showed a higher risk of not becoming pain free. Recurrence-free survival in 283 procedures was longer for PBC (44%) with 481 days compared to RFTC (56%) with 421 days (*p*=0.036) but without statistical significance. The only factors that showed a significant influence on longer recurrence-free survival rates were a postoperative BNI ≤ II (*P*=<0.0001) and a BNI facial numbness score ≥ 3 (*p* = 0.009). The complication rate of 22.2% as well as zero mortality showed no difference between the two procedures (*p*=0.162).

**Conclusion:**

Both percutaneous interventions led to a comparable initial pain relief and recurrence-free survival with a low and comparable probability of complications. An individualized approach, considering the advantages and disadvantages of each intervention, should guide the decision-making process. Prospective comparative trials are urgently needed.

## Introduction

Trigeminal neuralgia is a rare disease characterized by recurrent paroxysmal pain that lasts only for seconds to minutes in the distribution of one or multiple branches of the trigeminal nerve [3]. The International Headache Society classified trigeminal neuralgia based on the perceived cause into 1) classical trigeminal neuralgia due to a neurovascular conflict with morphological changes, 2) secondary trigeminal neuralgia due to another disease, e.g., tumor or multiple sclerosis, and 3) idiopathic trigeminal neuralgia with no or no compressive neurovascular conflict [18]. Another classification takes into account the phenotype of the disease, which differs between purely paroxysmal trigeminal neuralgia and trigeminal neuralgia with concomitant continuous pain [14]. First-line treatment consists of sodium channel blockers such as carbamazepine, followed by other anticonvulsive drugs and their combinations [3]. If drug therapy fails to relieve pain or is not tolerated by the patient, a plethora of surgical procedures may be offered. While microvascular decompression of the trigeminal nerve root should be the first choice in patients with classical and idiopathic trigeminal neuralgia, ablative procedures of the trigeminal ganglion have gained acceptance as minimally invasive techniques [4]. They may become useful in cases of idiopathic trigeminal neuralgia and secondary trigeminal neuralgia but also in classical trigeminal neuralgia if patients are not eligible for microvascular decompression or if microvascular decompression fails to relieve pain. Radiofrequency thermocoagulation (RFTC) and percutaneous balloon compression (PBC) are both relatively simple and well-established techniques that offer adequate pain relief combined with a good safety profile [11]. They mainly differ in the surgical method as well as in their specificity of nerve injury. While RFTC, popularized by Sweet and Wepsic in 1974, allows somatotopic nerve mapping and therefore selective lesioning of one or multiple divisions of the trigeminal nerve, it has to be performed while the patient is awake and cooperative [32]. In contrast, PBC, first described by Mullan and Lichtor in 1983, is less selective than RFTC but does not require an awake patient [25]. It is thought to selectively injure larger myelinated fibers and therefore is mainly useful in trigeminal neuralgia with pain in the ophthalmic division, as the small fibers of the corneal reflex may be spared [29]. While both techniques have already been evaluated alone or in comparison with other techniques, to the best of our knowledge, only a few studies have compared both methods with each other [15, 16, 26]. Therefore, we present our single-center experience comparing RFTC and PBC in patients with trigeminal neuralgia with regard to initial pain relief, recurrence-free survival and associated complications.

## Methods

### Patient cohort and study design

In this retrospective single-center study, we identified all patients who underwent PBC or RFTC of the trigeminal ganglion between January 2002 and December 2019 at the Medical University of Vienna to treat medically refractory trigeminal neuralgia. The diagnosis of trigeminal neuralgia was confirmed by reviewing the medical records and identifying typical neuralgia symptoms as defined by the International Headache Society [18]. Patients had to meet the following criteria for inclusion: 1) diagnosis of classical, idiopathic or secondary trigeminal neuralgia as defined by the European Academy of Neurology [3]; 2) age older than 18 years; and 3) complete medical record. Patients who had previous surgery for trigeminal neuralgia were included in the study. The study protocol was approved by the local ethics committee of the Medical University of Vienna (EK 1105/2020) and is in accordance with the Helsinki Declaration of Human Rights.

### Surgical procedures

**Fig. 1:**
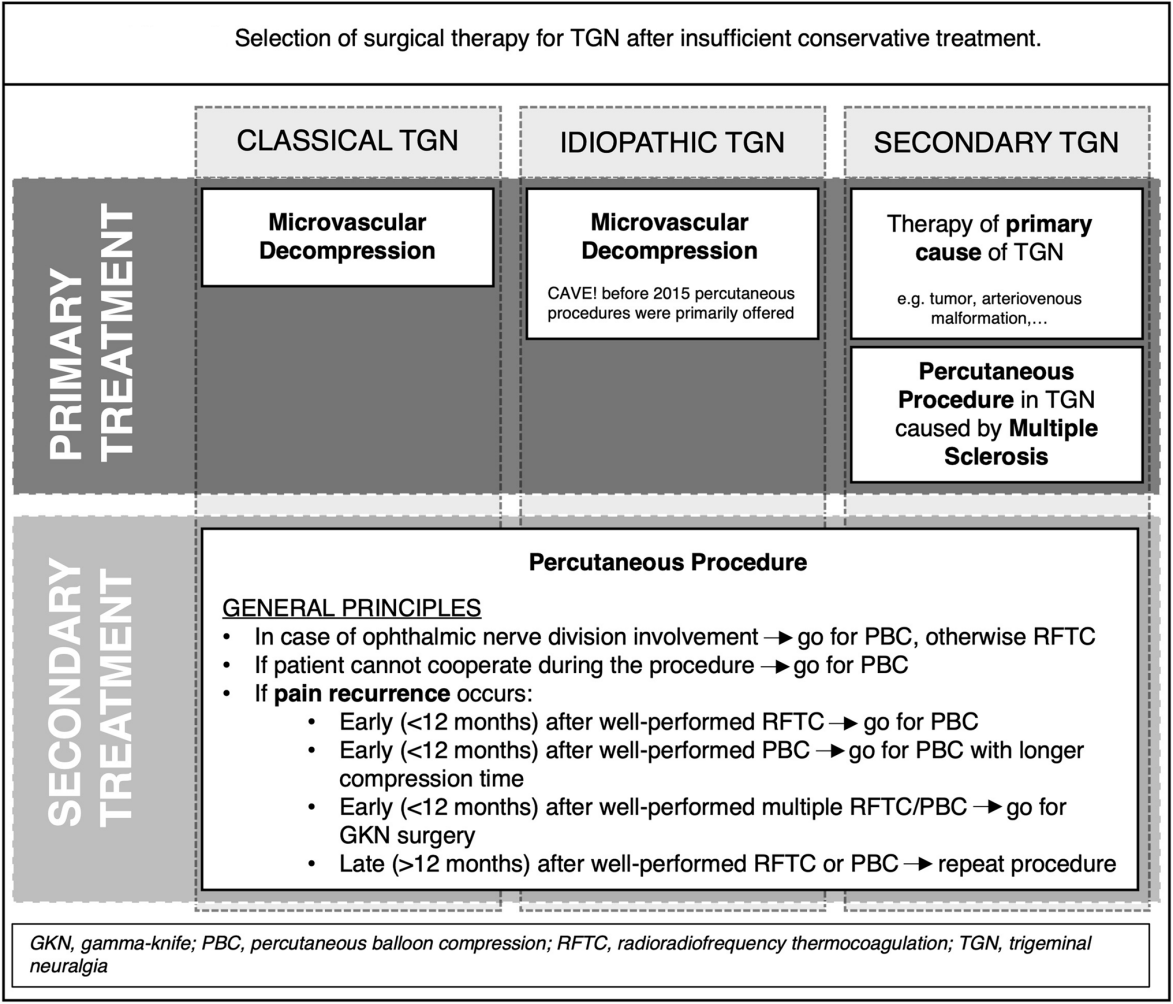
Selection of surgical therapy for trigeminal neuralgia after insufficient conservative treatment

The decision of whether to use PBC or RFTC to treat trigeminal neuralgia was made individually by one of three treating surgeons (Fig. 1). Factors influencing this decision were: the trigeminal division involved, the possibility of cooperating with the patient and the success of prior treatments. In general, patients with ophthalmic nerve involvement and patients who could not cooperate during an operation were primarily selected for PBC, while RFTC was preferred in patients with mandibular and maxillary involvement. If possible, the type of procedure was changed for follow-up interventions in the event of non-existent or only short-lasting therapeutic success. All operations were performed in the operating room. In PBC and RFTC, the same percutaneous approach to the foramen ovale was used as described by Härtel [17]. Patients were placed supine with their head in a reverse occipitomental position. A C-arm was used to allow fluoroscopic guidance. All patients underwent basic monitoring by electrocardiography, oxygen saturation and blood pressure measurements. After the procedure, patients were discharged the following day.

### Radiofrequency thermocoagulation

During RFTC, intravenous sedation and a nasopharyngeal airway were used. A 20-gauge standard radiofrequency lesioning needle with a straight 5 mm active tip (Radimed®) was inserted through the foramen ovale, positioning the needle tip in the trigeminal cistern as described by Tatli et al. [35]. Correct placement was confirmed by muscle contractions after motor stimulation (2 Hz, 0.1 ms). Having the patient responsive allowed fine needle adjustment according to the patients’ sensations during sensory stimulation (50 Hz, 1 ms). If paresthesia occurred in the affected division of the trigeminal nerve, patients received RFTC after the depth of anesthesia was adjusted. RFTC was performed at 70°C for 60 s. With the patient awake again, it was checked whether hypesthesia was present in the coagulated nerve division, which ended the procedure. If no hypesthesia was present, RFTC with the same settings was repeated once again after verifying the correct localization by stimulation. The needle was then removed, and the puncture side was held under pressure over five minutes.

### Percutaneous balloon compression

**Fig. 2:**
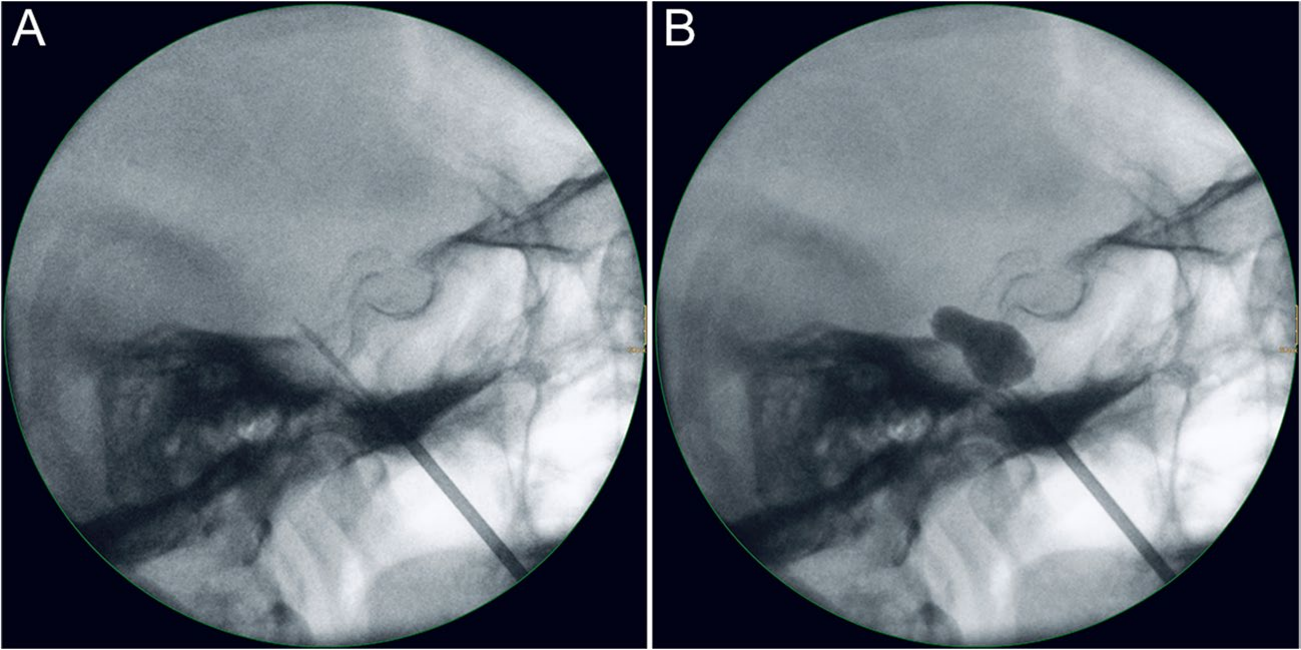
Fluoroscopy-guided percutaneous balloon compression in lateral view. **A**) The tip of the cannula slightly enters the foramen ovale, while the deflated Fogarty® embolectomy catheter is positioned with its tip at the border of the clivus. **B**) When the balloon is inflated, it ideally adopts the shape of the trigeminal cistern and reaches a pear-like shape with its tip lying in the trigeminal pore

An awake and cooperative patient is not required during PBC and therefore, general anesthesia was used. A 14-gauge cannula was inserted through the cheek and advanced just through the foramen ovale. The inner stylet was then removed. A 4 French Fogarty® embolectomy catheter was passed through the cannula and positioned in the trigeminal cistern with its tip at the trigeminal pore (Fig. 2, A). The balloon was then inflated twice with 0.7 ml of contrast agent (50%) and physiological saline solution (50%) over 60 s. Fluoroscopy confirmed adequate placement and shape of the balloon (Fig. 2, B). After compression, the cannula and the balloon were removed, and the puncture site was held under pressure over 5 min.

### Data collection and outcome measures

Data collection was obtained through patient admission charts, operative notes, preoperative MRT, discharge letters and follow-up reports. The clinical status of the patients was assessed by a neurosurgeon upon admission, discharge and during follow-up examinations. Since follow-up examinations in the outpatient clinic were not carried out in a standardized manner, all patients were contacted and summoned to the outpatient clinic for a personal follow-up visit for the sake of this study. Demographic data, trigeminal neuralgia symptoms including onset of trigeminal neuralgia, duration of symptoms, age at surgery, pain distribution, International Headache Society classification of trigeminal neuralgia (classic, idiopathic, secondary) and category of trigeminal neuralgia pain (purely paroxysmal vs. concomitant continuous), previous treatment, antiepileptic drugs and the Barrow Neurological Institute (BNI) pain intensity scale were recorded [30]. The BNI pain intensity scale classifies facial pain into I) no pain, no medication; II) occasional pain, not requiring medication; III) some pain, adequately controlled with medication; IV) some pain, not adequately controlled with medication; and V) severe pain/no pain relief [30].

Complications during and side effects after the procedures, such as intraoperative bradycardia, hypesthesia, corneal anesthesia, masseter muscle weakness, and anesthesia dolorosa, were noted for all cases, including those interventions that had to be canceled due to an anesthesiologic or a technical problem.

Initial pain relief was defined as a BNI of I, II and III with a one-point improvement in the first 4 weeks after surgery. Initial pain relief was documented for all patients who had undergone a successful procedure.

Recurrence of trigeminal neuralgia was defined as an increase in BNI after initial pain relief at the operated side. Recurrence-free survival was only calculated if patients had a follow-up of at least 6 months.

### Statistical analysis

Statistical calculations were carried out using GraphPad Prism version 9.1.2 (GraphPad Software, San Diego, California, USA) and R version 4.1.2 (R Foundation for Statistical Computing, Vienna, Austria). A two-sided *p*-value of < 0.05 was considered statistically significant. Continuous variables following a normal distribution were assessed by analysis of variance and Student’s *t*-test. Kruskal-Wallis and Mann-Whitney *U* tests were used for nonparametric interval-scaled variables. Chi-square or Fisher’s exact tests were used for categorical data. Multivariate logistic regression was used to identify individual variables that might influence initial pain relief after surgery. Kaplan-Meier survival analysis was performed with a time interval from surgery to the date of recurring pain and/or increase in antiepileptic medication. If pain was not recurrent, patients were censored at the last follow-up. Survival analysis was performed by using Mantel-Cox log-rank tests to identify individual variables associated with pain recurrence. Multivariate Cox proportional hazard regressions were used to quantify the effect of variables on recurrence-free survival.

## Results

**Fig. 3:**
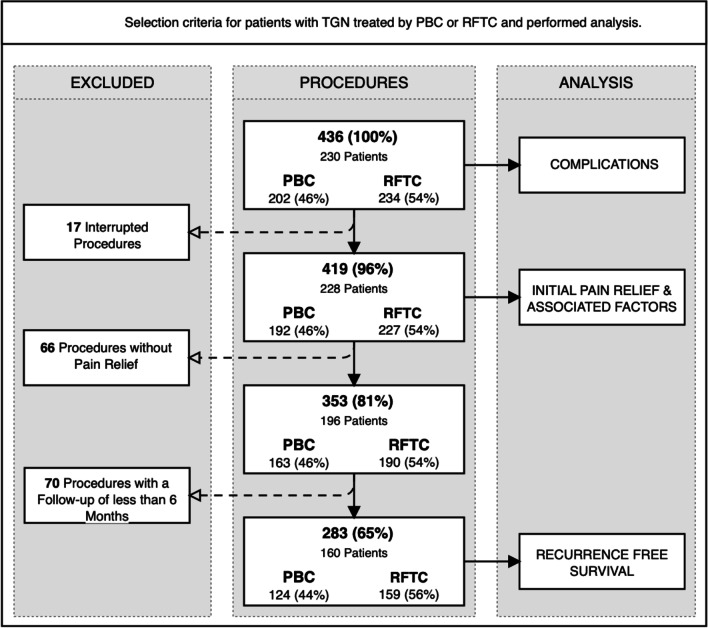
Selection criteria for patients with trigeminal neuralgia treated by percutaneous balloon compression or radiofrequency thermocoagulation and performed analysis

Eligible for the study were 230 patients who underwent 436 procedures with 202 (46%) PBC and 234 (54%) RFTC between 2002 and 2019. Seventeen procedures were terminated prior to ablation and excluded from the initial pain relief and recurrence-free survival analyses. A further 70 procedures had a follow-up shorter than six months and were excluded from the recurrence-free survival calculation (Fig. 3).

### Initial pain relief

Table 1 shows a detailed comparison between RFTC and PBC with regard to patient characteristics and initial pain outcome. In comparing the two procedures, a significant difference could only be seen for an older age at surgery (*p*=0.001), the operating time (*p*=<0.0001) and the presence of multiple sclerosis (*p*=0.015). Patients who underwent PBC had a higher median age of 70.5 years, shorter mean operating time of 18.1 ± 9.4 min and suffered less often from multiple sclerosis (30.7%) compared to 66 years, 33.3 ± 14.8 min and 42.7% in the RFTC group.

**Table 1:** Patient characteristics

	PBC	RFTC		All Patients
Variables	192 Procedures	227 Procedures	*p*-value	419 Procedures
Age at surgery (median, range), yr	70.5 (37-89)	66 (38-94)	0.001	68 (37-94)
Sex, n (%)			0.097	
Female	96 (50.0%)	133 (58.6%)		229 (54.7%)
Male	96 (50.0%)	94 (41.4%)		190 (45.3%)
Disease duration till surgery (mean ± SD), yr	10.8 ± 8	10.4 ± 10.4	0.602	10.6 ± 9.3
TGN classification, n (%)			0.083	
Classical	67 (34.9%)	61 (26.9%)		128 (30.5%)
Idiopathic	55 (28.6%)	60 (26.4%)		115 (27.4%)
Secondary	70 (36.5%)	106 (46.7%)		176 (42.0%)
Multiple sclerosis, n (%)			0.015	
Present	59 (30.7%)	97 (42.7%)		156 (37.2%)
Not Present	133 (69.3%)	130 (57.3%)		263 (62.8%)
Type of pain, n (%)			0.438	
Purely paroxysmal	156 (81.2%)	192 (84.6%)		348 (83.1%)
Concomitant continuous	36 (18.8%)	35 (15.4%)		71 (16.9%)
Affected side, n (%)			0.257	
Left	84 (43.8%)	113 (49.8%)		197 (47.0%)
Right	108 (56.2%)	114 (50.2%)		222 (53.0%)
Affected nerve branch, n (%)			-	
V1	1 (0.52%)	0 (0.00%)		1 (0.24%)
V1+V2	46 (24.0%)	5 (2.20%)		51 (12.2%)
V1+V2+V3	38 (19.8%)	6 (2.64%)		44 (10.5%)
V2	23 (12.0%)	56 (24.7%)		79 (18.9%)
V2+V3	68 (35.4%)	78 (34.4%)		146 (34.8%)
V3	16 (8.33%)	82 (36.1%)		98 (23.4%)
Baseline pain intensity, n (%)			0.342	
BNI II	1 (0.52%)	2 (0.88%)		3 (0.72%)
BNI III	1 (0.52%)	3 (1.32%)		4 (0.95%)
BNI IV	12 (6.25%)	24 (10.6%)		36 (8.59%)
BNI V	178 (92.7%)	198 (87.2%)		376 (89.7%)
Initial Pain Relief after surgery, n (%)			0.841	
No	29 (15.1%)	37 (16.3%)		66 (15.8%)
Yes	163 (84.9%)	190 (83.7%)		353 (84.2%)
Previous MVD, n (%)			0.781	
No	166 (86.5%)	193 (85.0%)		359 (85.7%)
Yes	26 (13.5%)	34 (15.0%)		60 (14.3%)
Operation time (mean ± SD), min	18.1 ± 9.4	33.3 ± 14.8	<0.001	26.1 ± 14.6
Number of previous operations (mean ± SD)	1.6 ± 1.9	1.7 ± 2.0	0.682	1.7 ± 2.0

*BNI*, Barrow Neurologic Institute; *MVD*, microvascular decompression; *PBC*, percutaneous balloon compression; *RFTC*, radiofrequency thermocoagulation; *TGN*, trigeminal neuralgia; *V1*, ophthalmic nerve; *V2*, maxillary nerve; *V3*, mandibular nerve

Initial pain relief after surgery was achieved in 353 (84.2%) procedures and was well balanced between the two groups, with initial pain relief rates of 84.9% for PBC and 83.7% for RFTC.

Multivariate logistic regression analyses showed that patients with multiple sclerosis and patients with a low preoperative BNI had worse initial pain relief with odds ratios of 5.53 and 1.99, respectively (Table 2).

**Table 2:** Potential risk factors for failed initial pain relief after PBC and RFTC

Risk factor	Odds ratio	95% confidence interval	*p*-value
Age at surgery	1.01	0.98 – 1.04	0.575
Procedure type [RFTC]	0.78	0.38 – 1.55	0.478
Fist ablative procedure [Yes]	0.84	0.38 – 1.84	0.665
Sex [Male]	1.09	0.59 – 2.03	0.775
Disease duration till surgery	1.02	0.98 – 1.06	0.388
Number of previous surgeries for TGN	0.87	0.70 – 1.10	0.230
Previous MVD	0.89	0.36 – 2.35	0.816
TGN classification [Idiopathic]	1.89	0.85 – 4.38	0.124
TGN classification [Secondary]	0.38	0.13 – 1.15	0.082
Multiple sclerosis TGN [Yes]	5.53	1.77 – 17.36	0.003
Type of pain [Purely paroxysmal]	1.43	0.68 – 2.88	0.328
Affected side [Left]	3.24	0.12 – 45.83	0.400
Affected side [Right]	5.15	0.19 – 72.41	0.241
Affected nerve branch V1	1.07	0.48 – 2.43	0.874
Affected nerve branch V2	0.69	0.30 – 1.53	0.375
Affected nerve branch V3	1.31	0.67 – 2.54	0.426
Preoperative BNI pain intensity scale	1.99	1.11 – 3.55	0.019
BNI facial numbness score 1+2*	0.98	0.51 – 1.88	0.954
BNI facial numbness score 3+4*	0.41	0.15 – 1.15	0.082

*BNI*, Barrow Neurologic Institute; *MVD*, microvascular decompression; *PBC*, percutaneous balloon compression; *RFTC*, radiofrequency thermocoagulation; *TGN*, trigeminal neuralgia; *V1*, ophthalmic nerve; *V2*, maxillary nerve; *V3*, mandibular nerve

*not fully documented in 177/419 procedures

### Pain recurrence

**Fig. 4:**
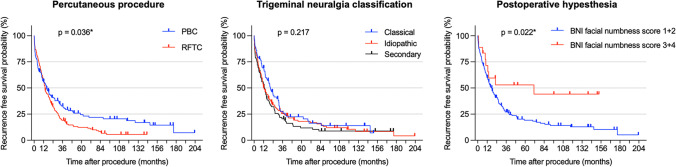
Kaplan-Meier survival analysis of patients with trigeminal neuralgia shows a slight advantage for percutaneous balloon compression over radiofrequency thermocoagulation in the probability of pain recurrence. Median pain recurrence was 481 days after percutaneous balloon compression in comparison to 421 days after radiofrequency thermocoagulation. While the classification of trigeminal neuralgia shows no influence, pronounced postoperative hypesthesia is likely to be a positive predictor on the duration of the postoperative pain-free interval. BNI, Barrow Neurologic Institute

Recurrence-free survival analysis was performed in 160 patients who underwent 283 (65%) procedures with a slight predominance for RFTC (56%) compared to PBC (44%) (Fig. 4). The median recurrence-free survival after PBC was not significantly longer (481 days) than that after RFTC (421 days), with a hazard ratio of 0.965 (Table 3). After one year, the recurrence rate was 56.1% for PBC and 56% for RFTC. Multivariate Cox proportional hazard regressions showed that the only factors that contributed to a significantly longer recurrence-free survival were a postoperative BNI pain intensity scale ≤ II (*p* = <0.001) and a BNI facial numbness score ≥ 3 (*p* = 0.009; Fig. 4). However, sufficient data on postoperative hypesthesia were only available in 176/283 (62.2%) procedures. Factors that were assumed to have an influence on recurrence-free survival such as: older age, longer duration of symptoms, concomitant continuous pain and the presence of multiple sclerosis showed no influence (Table 3). In Kaplan-Meier survival analysis, no advantage between PBC and RFTC could be seen in the treatment of multiple sclerosis patients (*p* = 0.103).

**Table 3:** Risk factors for pain recurrence after successful initial pain relief

Variables	No. procedures	Median recurrence free survival (d)	Hazard ratio	95% confidence interval	*p*-value
Procedure	283				
PBC	124	481			
RFTC	159	421	0.965	0.691 – 1.348	0.835
Sex					
Male	125	426			
Female	158	449	0.827	0.624 – 1.097	0.188
Age					
≤ 65 yr	130	428			
> 65 yr	153	434	0.896	0.644 – 1.247	0.516
Disease duration					
≤ 5 yr	88	394.5			
> 5 yr	184	434	0.859	0.621 – 1.190	0.362
* Not available*	*11*				
TGN classification					
Classical	72	625			
Idiopathic	82	370	1.265	0.869 – 1.841	0.219
Secondary	129	393	1.006	0.516 – 1.960	0.987
Multiple sclerosis					
MS present	117	370	1.221	0.651 – 2.292	0.534
MS not present	166	474			
Type of pain					
Purely paroxysmal	239	434			
Concomitant continuous	44	389	1.068	0.733 – 1.555	0.732
Affected side					
Left	132	363			
Right	151	543	0.934	0.701 – 1.246	0.644
Affected nerve branch					
V1 affected	56	323	0.918	0.623 – 1.354	0.666
V1 not affected	227	449			
Baseline BNI pain intensity scale					
≤ IV	26	255			
= V	257	454	0.652	0.406 – 1.046	0.076
Initial postoperative BNI pain intensity scale					
≤ II	60	2163			
> II	223	388	3.656	2.321 – 5.759	<0.001
Previous neurosurgery					
Previous MVD	39	603	0.756	0.475 – 1.202	0.237
First ablative surgery	110	449	0.878	0.475 – 1.202	0.501
> two surgeries	115	413	1.203	0.827 – 1.751	0.333
BNI facial numbness score 1+2*	158	530	0.766	0.572 – 1.025	0.073
BNI facial numbness score 3+4*	18	2163	0.377	0.181 – 0.787	0.009

*BNI*, Barrow Neurologic Institute; *MS*, multiple sclerosis; *MVD*, microvascular decompression; *PBC*, percutaneous balloon compression; *RFTC*, radiofrequency thermocoagulation; *TGN*, trigeminal neuralgia; *V1*, ophthalmic nerve

*not fully documented in 107/283 procedures

### Complications

We encountered an overall complication rate of 22.2% with a mortality rate of 0% (Table 4). The complication rate was slightly higher for PBC (25.3%) than for RFTC (19.7%) but did not reach significance (*p*=0.162). Surgery had to be terminated in 17 (3.9%) procedures because of 1) severe hemorrhage that was not suspended during the operation (1,6%), 2) the inability to correctly place the needle or the balloon (1,4%) and 3) the occurrence of an anesthesiologic problem related to the airway or the circulatory function regardless of the intervention (0.9%). Diplopia was encountered in 4 procedures but never in RFTC. Bradycardia commonly seen in ablative procedures had to be treated with medication in 60% of all cases (*n*=161; 36,9%). There was no case of corneal keratitis or anesthesia dolorosa after any percutaneous surgery. When analyzing risk factors for complications, there was no significant correlation with age (*p*=0.956), previous ablative procedures (*p*=0.102), ASA comorbidity score (0.411), or the presence of multiple sclerosis (0.351).

**Table 4:** Mortality, complications, and adverse events in 436 ablative surgeries

	PBC (*n*=202)	RFTC (*n*=234)	*p*-value	All procedures (*n*=436)
Mortality	0 (0%)	0 (0%)		0 (0%)
Complications				
Total	51 (25.3%)	46 (19.7%)	0.162	97 (22.2%)
Surgery discontinued	10 (5.0%)	7 (3%)	0.292	17 (3.9%)
Hemorrhage	5 (2.5%)	2 (0.9%)		7 (1.6%)
Anesthesiologic urgency*	1 (0.5%)	3 (1.2%)		4 (0.9%)
Technical failure	4 (2%)	2 (0.9%)		6 (1.4%)
Meningitis	3 (1.5%)	0 (0%)	0.099	3 (0.9%)
Hemorrhage	7 (3.5%)	5 (2.1%)	0.559	12 (2.8%)
Masticatory muscle weakness	4 (2.0%)	11 (4.7%)	0.186	15 (3.4%)
Diplopia	4 (2.0%)	0 (0%)	0.045	4 (0.9%)
Unwanted V1 hypesthesia	1 (0.5%)	1 (0.4%)	>0.999	2 (0.5%)
Paresthesia	7 (3.5%)	7 (3.0%)	0.793	14 (3.2%)
BNI facial numbness score ≥ III	15 (7.4%)	14 (6.0%)	0.569	29 (6.7%)
Arrhythmia during RFTC	0 (0%)	1 (0.4%)	>0.999	1 (0.2%)
Adverse Events				
Intraoperative bradycardia	96 (56.1%)	65 (32%)	0.0001	161 (36.9%)
no treatment required	31 (18.1%)	33 (16.3%)		64 (14.7%)
treatment required	65 (38%)	32 (15.8%)		97 (22.3%)
Balloon rupture	7 (3.4%)	-	-	7 (1.6%)

*, not related to the procedure; *BNI*, Barrow Neurologic Institute; *PBC*, percutaneous balloon compression; *RFTC*, radiofrequency thermocoagulation; *TGN*, trigeminal neuralgia; *V1*, ophthalmic nerve

## Discussion

PBC and RFTC are both minimally invasive techniques that offer good initial pain relief with a low-risk profile. The disadvantage comes with a significant recurrence rate that varies greatly between studies, ranging from 15 to 64% [1, 5, 9, 16, 20, 22, 27, 31, 34, 37, 39]. This large variation is partly attributed to differences in patient selection and institutional policies between centers. Comparative evaluations coming from a single center are scarce [15, 16, 26].

In our study, we showed that initial pain relief is comparable between PBC (84.9%) and RFTC (83.7%), while recurrence-free survival of PBC was found to be longer but not statistically significant in multivariate analysis, with a median recurrence-free survival of 481 days compared to 421 days after RFTC. After one year, the recurrence rate was 56.1% for PBC and 56% for RFTC. The recurrence rates given in the literature vary widely between 20% - 64% for PBC [5, 7, 9, 12, 22, 23, 31, 36] and 18–25% for RFTC [6, 16, 20, 36, 37].

This significant difference in the pain recurrence rate between our results and those reported in the literature can possibly be attributed to contrasting definitions of pain relief and recurrence, individual follow-up periods and varying compositions in the patient populations among centers. In our study, the definition of pain recurrence was set very strictly, meaning that every single pain attack and every increase in pain medication was already defined as a recurrence even if the patient improved again afterwards. Others, such as the study by Noorani et al., which showed a recurrence rate of only 25.4% for PBC and 38.2% for RFTC at 1 year, defined recurrence less strict as severe trigeminal pain not fully relieved by medication [26].

The patient population in our study was well balanced between both treatment groups with the exception of age, the presence of multiple sclerosis and the affected division of the trigeminal nerve, reflecting our treatment algorithm over the study period. Patients in the PBC group were significantly older, corroborating the fact that RFTC was performed on awake patients and testing in elderly patients may be complicated by potential cognitive impairment caused by sedation [28]. Likewise, patients with ophthalmic involvement were primarily selected for PBC to prevent corneal reflex impairment. As shown in animal studies, PBC preferentially affects medium and large myelinated fibers and spares small fibers [8]. In only 21/156 (13.5%) patients with multiple sclerosis the ophthalmic division was involved, explaining the high rate of multiple sclerosis patients among the RFTC group in our cohort.

### Risk factors for failed initial pain relief and recurrence

Multiple sclerosis and a low preoperative BNI pain intensity scale were found to be the only risk factors for a poor initial pain relief. The influence of the BNI with an odds ratio of 1.99 can easily be explained by the clearer defined difference between the preoperative and postoperative subjectively perceived pain in a very small group of patients (*n*=7 with a preoperative BNI pain intensity scale ≤ 3).

The dual pathology of multiple sclerosis and trigeminal neuralgia has been shown to have higher recurrence rates in multiple studies, and treatment failure occurs in most patients independently of the type of procedure [10, 24, 40]. While initial pain relief for patients with multiple sclerosis was poor in our study with an odds ratio of 5.53, no influence could be shown for recurrence-free survival. Furthermore, the chosen treatment modality did not show any influence in this subgroup of patients.

Mohammad-Mohammadi et al. showed that PBC (*n*=82) had the highest initial pain relief compared to RFTC (*n*=15), glycerol rhizolysis (*n*=89), microvascular decompression (*n*=10), stereotactic radiosurgery (*n*=52), and peripheral neurectomies (*n*=28). In their study, the median recurrence-free survival (initial procedure/repeat procedure) for PBC was 17/29 months and only 5/9 months for RFTC, which is comparable to the median recurrence-free survival rate of 370 days (≈12 months) in our study [24].

Patients with idiopathic trigeminal neuralgia had a higher but not significant initial failure rate than patients with classical trigeminal neuralgia. Furthermore, a trend towards earlier pain recurrence was also seen in this patient group. Why this patient group harbored poorer results is purely speculative, since the mechanism of damage is just not understood. Furthermore, it could be shown in surgical series that a large part (60%) of idiopathic trigeminal neuralgia defined by preoperative neuroimaging is likely also based on a vascular-nerve conflict [19].

Only a few studies have addressed predictive factors for long-term symptom improvement after PBC or RFTC. The only factors associated with a longer recurrence-free survival in our study were a low postoperative BNI pain intensity scale ≤ II and pronounced postoperative hypesthesia. Many studies anecdotally describe a positive correlation between postoperative hypesthesia and duration of pain relief [2, 26]. Reference is often made to a study by Taha et al. (1995), who performed thermal rhizotomy in 144 patients and who was able to correlate postoperative sensory loss with the rate of pain recurrence. In their cohort analgesia occurred in 46%, dense hypalgesia in 42% and mild hypesthesia in 12% of all patients with 14-year recurrence rates of 20%, 25% and 60%, respectively [33]. Due to the small number of patients in each group (postoperative BNI facial numbness score of 1, *n* = 20), the question if slight hypesthesia has a better recurrence-free survival than no postoperative hypesthesia cannot be seriously answered by our study. However, our data, as well as those available in the literature, allow the bothersome conclusion that a severely damaged trigeminal ganglion leads to a better recurrence-free survival.

While isolated third division pain has been shown to be a potential prognostic factor for a longer recurrence-free survival after RFTC, this could not be reproduced in our study [21]. Other subjective factors, such as a pear-shaped balloon during PBC, were not collected in our study [2, 22].

Furthermore, our study population shows a high number of patients needing follow-up surgeries (59.6%), secondary trigeminal neuralgia (42%) and concomitant continuous pain (16.9%). In all three groups, poorer initial pain relief and recurrence-free survival rates have been described in the literature, a finding that could, however, not be reproduced in our single-center series [10, 13, 26, 41].

### Complications

Both treatment modalities showed zero mortality and an overall complication rate of 25.3% for PBC and 19.7% for RFTC. While this overall complication rate seems high at first glance, one needs to take into account that paraesthesia and a BNI facial numbness score ≥ III were also counted as complications. Anesthesia dolorosa did not occur in any patient, and unwanted ophthalmic hypesthesia occurred in only two patients. The low rates of diplopia, masseter muscle weakness and meningitis are comparable with the current literature [38]. As most patients with ophthalmic division involvement undergo PBC, the rate of diplopia is lower in RFTC. The opposite is the case with masseter muscle weakness, which occurred primarily in patients after RFTC and mandibular division involvement.

Surgery had to be discontinued in 17 (3.9%) cases, of which 13 (2%) cases were related to the surgical procedure. Reasons for abundance were i) pronounced bleeding in the cheek area and ii) inability to enter the foramen ovale. Both complications are more common with PBC given the larger and therefore more traumatic cannula that needs to be used. Rare causes such as Eagle syndrome with an elongated styloid process and calcified stylohyoid ligament also occurred in our cohort. In these cases, technical adjuncts such as intraoperative navigation could help increase safety.

Intraoperative bradycardia occurred more frequently in PBC due to the trigeminal depressor response when entering through the foramen ovale with the larger needle or when inflating the balloon. This well-known phenomenon needs to be communicated with the anesthesiologist beforehand.

### Limitations

Like the majority of existing studies on percutaneous interventions for trigeminal neuralgia, this is a retrospective analysis with all the inherent known limitations. Potentially interesting factors that determined initial pain relief and recurrence-free survival in other studies could not be collected, such as the shape of the balloon, which was suggested to represent a key predictor of outcome in PBC [2, 22]. Furthermore, due to our selection criteria (Fig. 1) for one or the other treatment, there is an unavoidable selection bias and thus an unequal distribution in the treatment groups.

## Conclusions

Both percutaneous interventions led to a favorable and comparable initial pain relief in trigeminal neuralgia. In the long run, however, PBC was associated with a slightly but not significantly longer recurrence-free survival than RFTC. At present, there are no randomized controlled trials to seriously compare the effectiveness of ablative interventions. Based on the data currently available to us, both treatment modalities harbor distinct advantages and should be understood as concomitant rather than conflicting procedures. An individualized approach in a center able to offer all available modalities is warranted.

## Abbreviations

BNI: Barrow Neurological Institute
PBC: percutaneous balloon compression
RFTC: radiofrequency thermocoagulation
